# Putting in harm to cure: Drug related adverse events do not affect outcome of patients receiving treatment for multidrug-resistant Tuberculosis. Experience from a tertiary hospital in Italy

**DOI:** 10.1371/journal.pone.0212948

**Published:** 2019-02-28

**Authors:** Gina Gualano, Paola Mencarini, Maria Musso, Silvia Mosti, Laura Santangelo, Silvia Murachelli, Angela Cannas, Antonino Di Caro, Assunta Navarra, Delia Goletti, Enrico Girardi, Fabrizio Palmieri

**Affiliations:** 1 Respiratory Infectious Diseases Unit, National Institute for Infectious Diseases “L. Spallanzani” IRCCS, Rome, Italy; 2 Pharmacy Unit, National Institute for Infectious Diseases “L. Spallanzani” IRCCS, Rome, Italy; 3 Microbiology Unit, National Institute for Infectious Diseases "L. Spallanzani", IRCCS, Rome, Italy; 4 Clinical Epidemiology Unit, National Institute for Infectious Diseases "L. Spallanzani”, IRCCS, Rome, Italy; 5 Translational Research Unit, National Institute for Infectious Diseases "L. Spallanzani", IRCCS, Rome, Italy; Azienda Ospedaliera Universitaria di Perugia, ITALY

## Abstract

**Rationale:**

Treatment of multi-drug resistant Tuberculosis (MDR-TB) is challenging because it mostly relies on drugs with lower efficacy and greater toxicity than those used for drug-susceptible TB.

**Objectives:**

Aim of the study was to describe the frequency and type of adverse drug reactions in a cohort of MDR-TB patients and their potential impact on treatment outcome.

**Methods:**

We conducted a retrospective study in a cohort of MDR-TB patients enrolled at a tertiary referral hospital in Italy from January 2008 to December 2016. The records of patients were reviewed for epidemiological, clinical, microbiological and adverse drug reactions data.

**Results:**

Seventy-four MDR-TB patients (mean age 32 years, 58.1% males, 2 XDR, 12 pre-XDR TB) were extracted from the Institute data base and included in the retrospective study cohort in the evaluation period (January 2008—December 2016). Median length of treatment duration was 20 months (IQR 14–24). Treatment outcome was successful in 57 patients (77%; 51 cured, 6 treatment completed); one patient died and one failed (2.7% overall); 15 patients were lost to follow-up (20.3%). Sixty-six (89.2%) presented adverse drug reactions during the whole treatment period. Total number of adverse drug reactions registered was 409. Three hundred forty-six (84.6%) were classified as adverse events (AEs) and 63 (15.4%) were serious AEs (SAEs). One third of the total adverse drug reactions (134/409; 32.8%) was of gastrointestinal origin, followed by 47/409 (11.5%) ototoxic drug reactions, thirty-five (8.6%) regarded central nervous system and 33 (8.1%) affected the liver. All 63 SAEs required treatment suspension with 61 SAEs out of 63 (96.8%) occurring during the first six months of treatment. Factors associated with unsuccessful treatment outcome were smoking (p = 0.039), alcohol abuse (p = 0.005) and homeless condition (p = 0.044). Neither the number of antitubercular drugs used in different combinations nor the number of AEs showed significant impact on outcome. Patients who completed the treatment experienced a greater number of AEs and SAEs (p < 0.001) if compared to lost to follow-up patients.

**Conclusions:**

Our data demonstrate that, despite the high frequency of adverse drug reactions and long term therapy, the clinical management of MDR-TB patients in a referral center could reach successful treatment according to WHO target, by implementing active and systematic clinical and laboratory assessment to detect, report and manage suspected and confirmed adverse drug reactions.

## Introduction

Multidrug-Resistant Tuberculosis (MDR-TB), caused by *M*. *tuberculosis* strain resistant to both major drugs, isoniazid and rifampicin, remains a public health crisis and a health security threat [1]. Treatment of MDR-TB and extensively drug-resistant TB (XDR TB; MDR-TB with additional resistance to any fluoroquinolone, and to at least one of the three injectable second-line drugs amikacin, capreomycin or kanamycin) is challenging because it relies on drugs with lower efficacy and greater toxicity than those used for drug-susceptible TB (DS-TB) [2]. Treatment outcomes for MDR-TB and XDR TB are generally poor if compared to DS-TB [3]. In a systematic review conducted on 74 studies with 17,494 participants the proportion of treatment success in patients with MDR TB was 60% and only 26% in XDR-TB, with 17% of patients lost to follow-up globally [4]. Loss to follow-up is one of the main factors affecting unsuccessful outcome in MDR- and XDR-TB. Drug toxicity, long duration of treatment, and other social determinants have been associated with loss to follow-up [5]. Adverse drug reactions represent a potential obstacle to treatment completion and could negatively affect outcome [6]. Documenting, assessing and managing adverse drug events is important to achieve better patient compliance and improve treatment outcomes. Aim of the study was to describe frequency and type of adverse drug reactions in a cohort of MDR-TB patients and their potential impact on treatment outcome, especially lost to follow-up.

## Methods

Data on 74 MDR-TB patients hospitalized and followed at OPD from January 2008 to December 2016 at “L. Spallanzani” Hospital for Infectious Diseases in Rome have been extracted from the local database of TB patients and anonymized. WHO definition was adopted to define patient characteristics at diagnosis [7]. Respiratory samples collected from these patients underwent species identification and evaluation of resistance to rifampicin and isoniazid using the molecular assay GenoType MTBDRplus (Hain Lifescience, Germany), that are applied when there is a risk of MDR-TB. Furthermore, cultures isolated from these samples were subjected to susceptibility testing to first and second-line anti-tuberculous drugs. Drug-susceptibility testing procedures included testing on solid media (proportion method in Lowenstein-Jensen medium) and liquid media (MGIT 960 systems; Becton Dickinson, Sparks, MD, USA).

Patients were treated according to the institutional protocol, drawn up following WHO MDR-TB guidelines. An individualized regimen designed according to drug-susceptibility testing results and current WHO guidelines, adjusted for comorbidities, was used in all cases [8].

Initial treatment was provided on an in-hospital basis, until AFB sputum conversion was achieved on three consecutive negative samples collected during one week. Patients received Direct Observed Therapy during hospitalization. After discharge, patients were followed monthly on ambulatory care by trained MDR-TB specialists for the full course of treatment. Laboratory tests were repeated almost monthly, or as needed according to clinical condition, until the MDR-TB treatment was completed. Culture and direct examination of sputum smear were performed monthly, and at the end of treatment.

All patients received education about the administered drugs and duration of treatment. In particular, medical counseling was given about the possibility of adverse drug reactions occurrence and the importance of reporting any adverse event to their physician as soon as it presented. Patients were also provided with an information sheet about the most common side effects of anti-TB drugs. During each follow-up visit, patients were questioned about the occurrence of symptoms and treatment adherence.

Demographic and clinical data (such as age, gender, medical history and treatment regimen) were obtained by a researcher with experience in data management. Adverse events during treatment were collected by a trained researcher through review of medical records.

Treatment outcome of MDR-TB was defined according to WHO definition [7] and classified, for the purpose of this study, into two groups: successful outcome (cured and treatment completed) and unsuccessful outcome (treatment failed, died and lost to follow up/transferred out).

Adverse drug reactions were classified according to WHO definition in adverse events (AEs) and serious adverse events (SAEs). AE is any untoward medical occurrence that may present in a TB patient during treatment with a pharmaceutical product, but which does not necessarily have a causal relationship with this treatment. SAE is an AE that leads to: death or a life-threatening experience; hospitalization or prolongation of hospitalization; persistent or significant disability; or a congenital anomaly. SAEs may require a drastic intervention, such as termination of the drug suspected of having caused the event [9].

For adverse drug reactions that were confirmed by laboratory tests, an event was considered to occur if at least one laboratory value was abnormal. For adverse drug reactions not defined by laboratory tests, an event was considered to occur if physician documented the event in the patient chart according to clinical criteria and the patient-reported signs or symptoms. Adverse events were managed according to WHO guidelines [9].

The study was approved by Ethics Committee of National Institute for Infectious Diseases “L: Spallanzani” IRCCS with the decision n.12 (17^th^ of February 2015). All enrolled patients provided written informed consent to the utilization of anonymized clinical data.

### Statistical analysis

Descriptive analysis was conducted to characterize subjects enrolled in the study. Factors associated with different treatment outcome were assessed using Fisher’s exact test for categorical variables, and Mann-Whitney two-sample statistic to test differences for quantitative variables. All tests were two sided and p-values <0.05 were considered significant. Statistical analyses were conducted using *StataCorp*. *2013*. *Stata Statistical Software*: *Release 13*. *College Station*, *TX*: *StataCorp LP*.

## Results

A total of 74 MDR-TB patients were extracted from database and included in the retrospective study cohort in the period from January 2008 till December 2016.

Baseline characteristics of the 74 patients enrolled are shown in **Table 1.** The majority of patients (58.1%) were males. The median age of patients was 32 (IQR, 27–39) years. Three patients were HIV positive (4.1%). The number of drug resistances ranged from two to nine (median 5) with 43 patients (58.1%) having from 4 to 6. Two patients were XDR-TB and 12 were pre-XDR TB (MDR-TB with additional resistance to any fluoroquinolone, or to any of second-line injectable drugs).

**Table 1:** Characteristics of 74 TB-MDE Patients, 2008–2016.

Characteristics	N (%)
**Age at diagnosis–years**	median (IQR) 32 (27–39)
**Gender**	
Male	43 (58.1)
**Country of birth**	
Italy	9 (12.1)
East Europe	55 (74.3)
Asia	3 (4.1)
Africa	4 (5.4)
South America	3 (4.1)
**Employment status**	
Employed	33 (44.6)
Unemployed	33 (44.6)
Other	8 (10.8)
**Homeless**	
No	64 (86.5)
Yes	10 (13.5)
**IV drug user**	
No	63 (85.1)
Yes	5 (6.8)
Not known	6 (8.1)
**Smoker**	
Never	45 (60.8)
Current	15 (20.3)
Ex	2 (2.7)
Not known	12 (16.2)
**Alcohol abuse**	
Never	36 (48.6)
Current	17 (22.9)
Ex	3 (4.1)
Not known	18 (24.4)
**HIV status**	
Neg	71 (95.9)
Pos	3 (4.1)
**Diabetes**	
No	70 (94.6)
Yes	4 (5.4)
**Chronic hepatitis B or C**	
No	64 (86.5)
Yes	10 (13.5)
**Other Comorbidities**	
No	61 (82.4)
Yes	13 (17.6)
**BMI at admission**	
Underweight Normal Overweight	3 (4.1) 61 (82.4) 10 (13.5)
**Pattern of drug resistance**	
MDR	60 (81.1)
Pre-XDR	12 (16.2)
XDR	2 (2.7)
**Case definition**	
New case	42 (56.8)
Relapse	32 (43.2)
**Microscopy (respiratory samples) at referral**	
Negative	22 (29.7)
Positive	52 (70.3)
**Culture (respiratory samples)**	
Positive	74 (100)
**Localization**	
Pulmonary	69 (93.2)
Pulmonary + extra pulmonary	5 (6.8)
Extra pulmonary only	0
**Length of MDR-TB therapy (months)**	median (IQR) 20 (14–24)
≤6 months	11 (14.9)
7–18 months	18 (24.3)
≥19 months	45 (60.8)
**Year of TB diagnosis, class**	median (IQR) 20 (14–24)
2008–2010	19 (25.7)
2011–2013	27 (36.5)
2014–2016	28 (37.8)
**Outcome**	
Cured	57 (77.0)
Dead/failure	2 (2.7)
LTFU/Transferred	15 (20.3)

IQR: Inter quartile range

**Fig 1:**
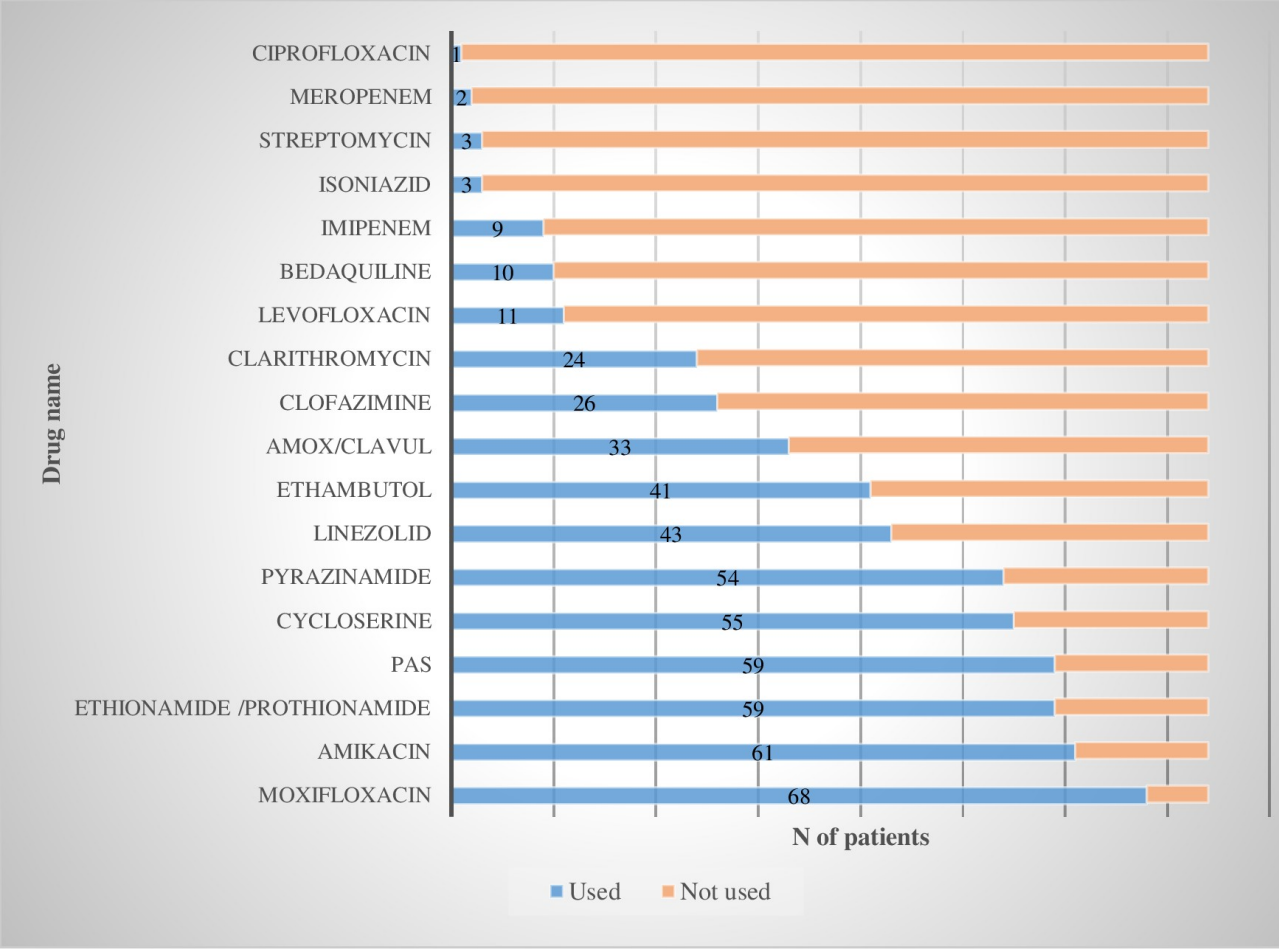
Regimen composition of 74 patients with MDR TB.

MDR-TB cases in this cohort received a mean of 7 (6–9) anti-tuberculosis drugs all over the course of treatment. In **Fig 1** drugs prescribed are listed by name.

Median length of treatment duration was 20 months (IQR 14–24); the drug exposition period was inferior to six months in 11 lost to follow up patients (14.8%).

Treatment outcome was successful in 57 patients (77%; 51 cured and 6 treatment completed); one patient died and one failed (2.7% overall); 15 patients were lost to follow-up (20.3%).

Sixty-six out of 74 patients (89.2%) presented AEs during the whole treatment period.

Total number of adverse drug reaction registered was 409. Three hundred forty-six (84.6%) were AEs and 63 (15.4%) were SAEs.

Thirty-seven patients (50%) presented one or more AEs; twenty-seven (36.5%) presented at least one SAEs with one or more AEs and 2 patients (2.7%) only SAEs.

In 27 out of 29 patients (93.1%) SAEs were recorded in the first 6 months of treatment **(Table 2)**.

**Table 2:** Number of AEs and SAEs per apparatus and clinical management.

Apparatus	AEs	SAEs in first six months of treatment A B C			Total SAEs*	Total AEs and SAEs
Gastrointestinal	**111**	**0**	**0**	**23**	**23**	**134**
Ear	**31**	**0**	**15**	**1**	**16**	**47**
Central Nervous System	**32**	**0**	**2**	**1**	**3**	**35**
Liver	**22**	**0**	**2**	**9**	**11**	**33**
Skin	**28**	**0**	**0**	**3**	**3**	**31**
Metabolism	**30**	**0**	**0**	**0**	**0**	**30**
Kidney	**24**	**0**	**0**	**0**	**0**	**24**
Peripheral Nervous System	**17**	**1**	**1**	**0**	**3**	**20**
Bone	**18**	**0**	**0**	**0**	**1**	**19**
Endocrine	**17**	**0**	**0**	**1**	**1**	**18**
Blood	**8**	**0**	**1**	**1**	**2**	**10**
Respiratory	**5**	**0**	**0**	**0**	**0**	**5**
Cardiovascular	**3**	**0**	**0**	**0**	**0**	**3**
Total	**346**	**1**	**21**	**39**	**63**	**409**

A: the drug was suspended; B: the drug was suspended and one or two drugs were added; C: the drug was resumed after suspension

* SAEs during whole treatment period (including 2 episodes after 6 months)

One third of the total adverse drug reactions (134/409; 32.8%) was of gastrointestinal origin (111 AEs; 23 SAEs), followed by 47/409 (11.5%) adverse drug reactions of the ear (31 AEs; 16 SAEs), thirty-five (8.6%) regarded central nervous system (32 AEs; 3 SAEs) and 33 (8.1%) the liver (22 AEs; 11 SAEs). These four groups cover the majority of the total burden of adverse drug reactions (249/409; 61%). Other adverse drug reactions reported affected metabolism (7.3%), renal function (5.9%), peripheral nervous system (4.9%), endocrine (4.4%) and hematopoietic function (2.4%).

When we analyzed the association of specific AEs with specific drugs, we did not found a statistically significant association with the majority of complaints, even because in most of cases patients were taking more than one drug able to cause similar AEs, as well known for gastrointestinal symptoms.

Injectable agents was the most common single drug withdrawn, especially during the first 6 months of treatment. Twenty-six out of 63 patients (41.3%) experienced hypoacusia: 23 patients exposed to Amikacin, and 3 exposed to Streptomycin. Among the other 11 MDR-TB patients not exposed to injectable drugs, only one presented hearing disturbances.

Linezolid (daily dose 600 mg) was part of treatment for 43 patients and 14 of them (32.6%) developed peripheral neuropathy. Among 31 patients that did not assume linezolid, only 4 (12.9%) developed peripheral neuropathy, probably associated to cycloserine.

In our country bedaquiline became accessible for compassionate use at the end of 2014, and we treated 10 patients in this cohort. As a whole, they had 49 adverse drug reactions during antitubercular treatment, of whom 14 were SAEs. None among ten patients treated with bedaquiline for six months had QTc interval more than 450 ms. Noteworthy two patients out of 68 (2.9%) treated with fluoroquinolones presented QTc prolongation between 450 and 500 ms.

AEs and SAEs were managed according to WHO guidelines [9], as described in **S1 Table (S1 Table. AE and SAE per apparatus and clinical management).**

AEs were generally managed with supportive treatment and in no case required treatment suspension.

All 63 SAEs required treatment suspension with 61 out of 63 SAEs (96.8%) happening during the first six months of treatment. In 39 out of 63 episodes of SAEs (62%) the drug held responsible was resumed after discontinuation. Time of suspension in case of reaction was variable, according to severity of AEs and clinical conditions of patients.

Rechallenge with full dose upfront was done in all cases of SAEs except for rash and DRESS, in which case, after resolving, drugs are reintroduced one at a time with incremental dose, starting from the less suspected one. Drug rechallenge was operated mostly after recovery from adverse events of gastrointestinal type (23/39; 59%), followed by liver (9/39; 23.1%) and dermatological (3/39; 7.7%). Rechallenge was possible also in one case of blood, endocrine, ear and central nervous system SAEs respectively (4/39; 10%).

In 23 out of 63 SAEs (36.5%) one or two other drugs were added after suspension of suspected drug; in one case the drug was suspended and no other drug was added. **See S1 Table.**

The reintroduction of drug was not possible in 2 out 2 cases of drug induced severe peripheral neuropathy and in all 15 cases of severe hearing loss. In SAEs involving central nervous system the drug was not resumed in 2 out of 3 cases (66.6%).

The univariate analysis on factors influencing treatment outcome found that women were at lower risk of unsuccessful outcome (p = 0.026). Factors associated with unsuccessful treatment outcome were smoking (p = 0.039), alcohol abuse (p = 0.005) and homeless condition (p = 0.044). Number of antitubercular drugs used in different combinations, pattern of drug resistance and adverse drug reactions did not show significant impact on outcome. Patients who completed the treatment experienced a higher number of adverse drug reactions (p < 0.001) compared to lost to follow-up patients **(Table 3**).

**Table 3:** Characteristics of 74 patients: 57 successful vs 17 unsuccessful outcome.

Characteristics	Successful (N = 57)	Unsuccessful (N = 17)	Total (N = 74)	p-value
**Age at diagnosis**,years				0.475*
(median IQR)	32 (26,5–38)	35 (28,5–41,5)	32 (27–39)	
**Gender**				0.026
Male	29 (67.4%)	14 (32.6%)	43 (100%)	
Female	28 (90.3%)	3 (9.7%)	31 (100%)	
**Origin**				0.107
Italy	9 (100%)	0	9 (100%)	
Foreign born	48 (73.8%)	17 (26.2%)	66 (100%)	
**Homeless**				0.044
No	52 (81.2%)	12 (18.8%)	64 (100%)	
Yes	5 (50.0%)	5 (50.0%)	10 (100%)	
**IDU**				0.840
No	49 (77.8%)	14 (22.2%)	63 (100%)	
Yes	4 (80.0%)	1 (20.0%)	5 (100%)	
Unknown	4 (66.7%)	2 (33.3%)	6 (100%)	
**Smoking status**				0.039
Never	39 (86.7%)	6 (13.3%)	45 (100%)	
Current or ex	10 (58.8%)	7 (41.2%)	17 (100%)	
Unknown	8 (66.7%)	4 (33.3%)	12 (100%)	
**Alcohol abuse**				0.005
Never	33 (91.7%)	3 (8.3%)	36 (100%)	
Current or ex	11 (55.0%)	9 (45.0%)	20 (100%)	
Unknown	13 (72.2%)	5 (27.8%)	18 (100%)	
**HIV status**				1.000
Negative	54 (76.1%)	17 (23.9%)	71 (100%)	
Positive	3 (100%)	0	3 (100%)	
**Diabetes**				1.000
No	54 (77.1%)	16 (22.9%)	70 (100%)	
Yes	3 (75.0%)	1 (25.0%)	4 (100%)	
**Chronic hepatitis (B or C)**				0.669
No	51 (79.7%)	13 (20.3%)	64 (100%)	
Yes	6 (60.0%)	4 (40.0%)	10 (100%)	
**BMI at admission**				0.224
Underweight	2 (66.7%)	1 (33.3%)	3 (100%)	
Normal	47 (77.0%)	14 (23.0%)	61 (100%)	
Overweight	8 (80.0%)	2 (20.0%)	10 (100%)	
**Case Definition**				0.169
New case	35 (83.3%)	7 (16.7%)	42 (100%)	
Relapse/ Chronic/Failure	22 (68.7%)	10 (31.3%)	32 (100%)	
**Treatment duration**, months				<0.001
Less or equal 6 months	0	11 (100%)	11 (100%)	
Between 7–18 months	14 (77.8%)	4 (22.2%)	18 (100%)	
More than 18 months	43 (95.6%)	2 (4.4%)	45 (100%)	
**Number of Drugs used**				0.738*
(median IQR)	7 (6–9)	7 (6–8)	7 (6–9)	
**Pattern of drug resistance**				1.000
MDR	46 (76.7%)	14 (23.3%)	60 (100%)	
XDR/pre-XDR	11 (78.6%)	3 (21.4%)	14 (100%)	
**TB localization**				1.000
Pulmonary	53 (76.8%)	16 (23.2%)	69 (100%)	
Pulmonary + extra pulmonary	4 (80.0%)	1 (20.0%)	5 (100%)	
**Year of diagnosis**				0.779
2008–2010	16 (84.2%)	3 (15.8%)	19 (100%)	
2011–2013	20 (74.1%)	7 (25.9%)	27 (100%)	
2014–2016	21 (75.0%)	7 (25.0%)	28 (100%)	
**Adverse Events**				0.665
None	5 (62.5%)	3 (37.5%)	8 (100%)	
AE (one or more)	28 (75.7%)	9 (24.3%)	37 (100%)	
SAE First 6 months	22 (81.5%)	5 (18.2%)	27 (100%)	
SAE First 6 months and after	2 (100%)	0	2 (100%)	

IQR: Inter quartile range

*Mann-Whitney’s test

Fifteen cases out of 74 (20.3%) interrupted the treatment and were lost to follow up (LTFU). All these patients were foreigners and most of them (10/15; 67%) were unemployed and/or homeless; eight out of 15 were also alcohol abusers. In the analysis performed, considering LTFU and dead/failure separately, some social conditions (unemployment and homelessness) and alcohol abuse were more frequent in LTFU group compared to the cured patients while there was no higher incidence of AEs and SAEs in LTFU patients (**S2** and **S3 Tables; S2 Table. Correlation between outcome and social constraints; S3 Table. Correlation between outcome and AEs/SAEs).**

## Discussion

We found that adverse drug reactions during MDR-TB treatment were common and they did not significantly affect outcome.

Sixty-six out of 74 patients (89.2%) presented 409 adverse drug reactions (346 AEs and 63 SAEs) during the whole treatment period. Sixty-one out of 63 SAEs (96.8%) occurred during the first 6 months of treatment.

Documenting, assessing and managing adverse drug events is important to achieve better patient compliance and improve treatment outcomes. Active TB drug-safety monitoring is a recent introduction in TB programs and its implementation is still ongoing in many countries [10]. There is no global consensus on the overall incidence of adverse drug reactions related to MDR-TB treatment. In a recent systematic review including 17,494 subjects Bastos and coll. reported SAEs ranging from less than 1% to more than 10% with each drug [4], but, as Authors stated, bias due to underreporting of adverse drug reactions could have influenced results. Data reported in our study are consistent with 79% of incidence of adverse drug reactions reported by Bloss and coll. in Latvia [11], 90%, Zhang and coll. in a Chinese population [12] and 77% incidence reported in the study from Dela and coll. in India [13].

As previously reported from Wu and coll. [14] we found that in 27 patients (36.5%) SAEs were recorded in the first 6 months of treatment. Consistently with data published hearing loss was a concern, 15/74 patients (20,3%) experienced hearing loss during the first six months of treatment and the most common permanently discontinued drug was injectable (Amikacin) [11–14]. Hearing loss is a well-known threat occurring in MDR-TB patients and the use of injectable drugs is under discussion in favor of more tolerated drugs [15]. Based on our experience hearing loss should be carefully and actively exanimated, in order to avoid severe hearing loss.

We found that in all cases of linezolid-associated severe peripheral neuropathy the drug was suspended. Our incidence of SAEs due to linezolid (2/63; 3.2%) was lower than 58.9%, as reported by Sotgiu and coll. in a systematic review [16].

The prevalence of other AEs and SAEs by apparatus in our study was similar to data reported by other authors [11–13; 17]. Gastrointestinal symptoms were very frequent (32.8% of adverse drug reactions), consistent with data published by Furin and coll. with 100% of patient reporting almost mild gastritis [18].

Adverse drug reactions involving central nervous system were also common (25% of patients) and the frequency found was similar to 25% reported from Trebucq and coll [19] and 21.5% reported from Avong [20]. SAEs involving CNS and attributed to cycloserine were registered only 3 times (4.8% of events) with same prevalence of 4.5% estimated from Bastos and coll. [4].

During the period of study there were several changes in MDR-TB regimen recommendations. Nevertheless, major drugs in regimen composition did not change until Bedaquiline became available.

Noteworthy no severe QTC prolongation (> 500 ms) has been observed, in fluoroquinolones and especially bedaquiline treated patients, confirming data on low incidence of cardiac SAEs in patients receiving drugs known for QTC prolongation risk [21]. Only two patients of the cohort presented QTc prolongation (between 450 and 500ms), that was associated to Fluoroquinolones.

As well known, repeated adverse drug reactions lead to changes in regimen that could have impact on treatment success. As described in **Table 2**, in our series of 63 SAEs cases (15.4%) there was a temporary or permanent suspension of suspected drug compared to 30–56% of cases in other studies of MDR-TB cohorts [22].

Frequent patients monitoring and prompt intervention on symptoms and adverse drug reactions during the early months of treatment is a fundamental part of MDR-TB management [9]. In fact, in our study 96.8% of SAEs were recorded in the first 6 months of treatment. Patient’s education about possible side effects and query using a standardized approach at each clinical evaluation help to successfully complete treatment, reducing the risk of adverse drug reactions, even severe ones [23]. It is important that the patient understands the importance of each medication, the possible adverse drug reactions and what to do when they occur. In our data the duration of treatment, while improving the chance to be cured, poses the threat for adverse drug reactions occurring (p <0.001; **Table 3**). Even if results could be attributable to bias, our data are consistent with published reports showing that a longer period of treatment confers higher chance to be cured although with more adverse drug reactions [24]. In our series we found that tailored drug regimens allowed rates of treatment success in a mixed population of MDR- and XDR-TB patients of 77%, corresponding to the target proposed by WHO [6]. Noteworthy the two XDR-TB patients completed treatment and were judged cured (100% treatment success). We observed one case of death (1.35%) that is a lower percentage of mortality than reported from other series, accounting for 14% as recently reported [15].

The worrisome rate of patients lost at follow up in our series deserves attention.

TB patient’s lost to follow-up are more likely to develop infectious active TB again, contributing to maintain burden of disease, especially in a low prevalence country like Italy, representing an obstacle to END TB targets [25]. Previous research conducted in many different contexts suggests that MDR-TB treatment outcomes are influenced by factors specific to individual patients and to local TB treatment programs [26].

In our cohort the group of LTFU was consistent (15/74; 20.3%). Unfavorable social conditions were more frequent in the group of LTFU compared to the cured patients, while there was no significant difference between the two groups in AEs and SAEs incidence. It is possible to hypothesize that social constraints and alcohol abuse negatively influence the ability of patients to tolerate a long and difficult treatment, burdened by many side effects.

Even in Italy, where there is a national health system providing universal coverage for hospital and ambulatory care of TB, our data confirm that homeless condition and risk behaviors are associated to unsuccessful outcome of MDR-TB treatment. In our study alcohol consumption is a risk factor for unsuccessful outcome, consistent with several studies reporting that persons who drink excessively are more likely to have treatment interruptions [27].

Limited experience in MDR-TB care is likely to result in inappropriate clinical case management and emergence of resistance. As suggested by European Centre for Diseases Prevention and Control, concentration of in-patient MDR-TB treatment could be considered in countries with low numbers of MDR-TB patients [28]. Individualized management of patients at a tertiary level center may have played an important role in the achievement of high rates of treatment success. The use of drug combinations adapted to resistance profiles, active drug safety monitoring during the full treatment course, and the continuous availability of second-line and new anti-TB drugs were all key factors [29].

Major limitation of this study is due to its retrospective nature and to the small sample size: all of the source records and reports were not designed for study purposes and some information could be incomplete. Our data do not allow to determine the adverse drug reactions of a single drug and underreporting of adverse drug reactions could have influenced study results.

In conclusion, our data demonstrate that, despite facing a high frequency of adverse drug reactions and long term therapy, MDR-TB patients can benefit from a careful clinical management that implements drug safety monitoring. In our referral center, active and systematic clinical and laboratory assessment to detect, report and properly manage suspected and confirmed adverse drug reactions ensured to achieve WHO target treatment success rates. Addressing social determinants associated to low adherence to TB cure represents an urgent target for intervention in order to diminish obstacles to treatment completion.

## Supporting information

S1 Table AE and SAE per apparatus and clinical management. (DOCX)

S2 Table Correlation between outcome and social constraints. (DOCX)

S3 Table Correlation between outcome and AEs/SAEs. (DOCX)
