# Supplementary material for: Putting in harm to cure: Drug related adverse events do not affect outcome of patients receiving treatment for multidrug-resistant Tuberculosis. Experience from a tertiary hospital in Italy
Source: PLoS One. 2019 Feb 28;14(2):e0212948. doi: 10.1371/journal.pone.0212948 (PMC6394924; doi:10.1371/journal.pone.0212948)
Supplement: S1 Table — (DOCX) [file pone.0212948.s001.docx]

**Supporting information tables**

**S1 Table. AE and SAE per apparatus and clinical management**

| **Apparatus** | **Adverse Event** |  |  | **SAE*** | | |  |  |
| --- | --- | --- | --- | --- | --- | --- | --- | --- |
|  |  | **AE** | **A** | **B** | **C** | **D** | **P** | **Total** |
| **GASTROINTESTINAL** | Gastric ulcer | 2 | 0 | 0 | 0 | 0 | 0 | 2 |
|  | Epigastric pain | 10 | 0 | 0 | 0 | 0 | 0 | 10 |
|  | Abdominal pain | 8 | 0 | 0 | 0 | 1 | 0 | 9 |
|  | Dyspepsia | 23 | 0 | 0 | 0 | 5 | 0 | 28 |
|  | Nausea | 24 | 0 | 0 | 0 | 6 | 0 | 30 |
|  | Vomiting | 17 | 0 | 0 | 0 | 6 | 0 | 23 |
|  | Diarrhoea | 12 | 0 | 0 | 0 | 5 | 0 | 17 |
|  | Appetite loss | 15 | 0 | 0 | 0 | 0 | 0 | 15 |
|  | **Total** | **111** | **0** | **0** | **0** | **23** | **0** | **134** |
| **EAR** | Hearing loss | 12 | 0 | 12 | 3 | 0 | 0 | 27 |
|  | Tinnitus | 7 | 0 | 0 | 0 | 0 | 0 | 7 |
|  | Vertigo | 6 | 0 | 0 | 0 | 0 | 0 | 6 |
|  | Dizziness | 4 | 0 | 0 | 0 | 1 | 0 | 5 |
|  | Otitis media | 2 | 0 | 0 | 0 | 0 | 0 | 2 |
|  | **Total** | **31** | **0** | **12** | **3** | **1** | **0** | **47** |
| **CENTRAL NERVOUS SYSTEM** | Depression/Suicidal ideas | 18 | 0 | 2 | 0 | 1 | 0 | 21 |
|  | Anxiety | 12 | 0 | 0 | 0 | 0 | 0 | 12 |
|  | Headache | 2 | 0 | 0 | 0 | 0 | 0 | 2 |
|  | **Total** | **32** | **0** | **2** | **0** | **1** | **0** | **35** |
| **LIVER** | AST/ALT increase | 15 | 0 | 0 | 1 | 6 | 0 | 22 |
|  | Symptomatic hepatotoxicity | 7 | 0 | 0 | 1 | 3 | 0 | 11 |
|  | **Total** | **22** | **0** | **0** | **2** | **9** | **0** | **33** |
| **SKIN** | Maculopapular rash | 7 | 0 | 0 | 0 | 1 | 0 | 8 |
|  | Dermatitis | 2 | 0 | 0 | 0 | 0 | 0 | 2 |
|  | Skin pigmentation | 13 | 0 | 0 | 0 | 0 | 0 | 13 |
|  | Photosensitivity | 1 | 0 | 0 | 0 | 0 | 0 | 1 |
|  | DRESS | 0 | 0 | 0 | 0 | 2 | 0 | 2 |
|  | Pain in injection site | 2 | 0 | 0 | 0 | 0 | 0 | 2 |
|  | Oedema | 2 | 0 | 0 | 0 | 0 | 0 | 2 |
|  | Recurrent Candida infection | 1 | 0 | 0 | 0 | 0 | 0 | 1 |
|  | **Total** | **28** | **0** | **0** | **0** | **3** | **0** | **31** |
| **METABOLISM** | Weight loss | 15 | 0 | 0 | 0 | 0 | 0 | 15 |
|  | Astenia | 14 | 0 | 0 | 0 | 0 | 0 | 14 |
|  | Hypercholesterolemia | 1 | 0 | 0 | 0 | 0 | 0 | 1 |
|  | **Total** | **30** | **0** | **0** | **0** | **0** | **0** | **30** |
| **KIDNEY** | Hyperuricemia | 12 | 0 | 0 | 0 | 0 | 0 | 12 |
|  | Proteinuria | 3 | 0 | 0 | 0 | 0 | 0 | 3 |
|  | Hypokalaemia | 1 | 0 | 0 | 0 | 0 | 0 | 1 |
|  | Hyperkalaemia | 1 | 0 | 0 | 0 | 0 | 0 | 1 |
|  | Hypomagnesaemia | 1 | 0 | 0 | 0 | 0 | 0 | 1 |
|  | Renal impairment | 6 | 0 | 0 | 0 | 0 | 0 | 6 |
|  | **Total** | **24** | **0** | **0** | **0** | **0** | **0** | **24** |
| **PERIPHERAL NERVOUS SYSTEM** | Peripheral neuropathy | 16 | 0 | 0 | 1 | 0 | 1 | 18 |
|  | Blurring vision | 1 | 1 | 0 | 0 | 0 | 0 | 2 |
|  | **Total** | **17** | **1** | **0** | **1** | **0** | **1** | **20** |
| **BONE** | Arthralgia | **18** | **0** | **0** | **0** | **0** | **1** | **19** |
| **ENDOCRINE** | Hypo-thyroidism | 16 | 0 | 0 | 0 | 1 | 0 | 17 |
|  | Amenorrhea | 1 | 0 | 0 | 0 | 0 | 0 | 1 |
|  | **Total** | **17** | **0** | **0** | **0** | **1** | **0** | **18** |
| **BLOOD** | Neutropenia | 2 | 0 | 0 | 0 | 1 | 0 | 3 |
|  | Leucopenia | 2 | 0 | 0 | 0 | 0 | 0 | 2 |
|  | Anemia | 4 | 0 | 1 | 0 | 0 | 0 | 5 |
|  | **Total** | **8** | **0** | **1** | **0** | **1** | **0** | **10** |
| **RESPIRATORY** | Dispnea | **5** | **0** | **0** | **0** | **0** | **0** | **5** |
| **HEART** | Palpitation | 1 | 0 | 0 | 0 | 0 | 0 | 1 |
|  | QTc prolongation (450-500) | 2 | 0 | 0 | 0 | 0 | 0 | 2 |
|  | QTc prolongation (> 500) | 0 | 0 | 0 | 0 | 0 | 0 | 0 |
|  | **Total** | **3** | **0** | **0** | **0** | **0** | **0** | **3** |
|  |  |  |  |  |  |  |  |  |
| **Total adverse events reported** | | **346** | **1** | **15** | **6** | **39** | **2** | **409** |

A: the drug was suspended; B: the drug was suspended and another drug was added; C: the drug was suspended and two drugs were added; D: the drug was resumed after suspension; P: SAE that happened after 6 months of treatment
