# Supplementary material for: Putting in harm to cure: Drug related adverse events do not affect outcome of patients receiving treatment for multidrug-resistant Tuberculosis. Experience from a tertiary hospital in Italy
Source: PLoS One. 2019 Feb 28;14(2):e0212948. doi: 10.1371/journal.pone.0212948 (PMC6394924; doi:10.1371/journal.pone.0212948)
Supplement: S2 Table — (DOCX) [file pone.0212948.s002.docx]

**Supporting information tables**

**S2 Table. Correlation between outcome and social constraints**

| **Outcome** | **N.** | ***Unemployed*** | ***Homeless*** | ***Alcohol abuse*** |
| --- | --- | --- | --- | --- |
| **Cured** | 57 | 22 (38.6%) | 5 (8.8%) | 11 (19.3%) |
| **LTFU** | 15 | 10 (66.7%) | 4 (26.7%) | 8 (53.3%) |
| **Dead/ failure** | 2 | 1 (50%) | 1 (50%) | 1 (50%) |
| **p-value** |  | 0.284 | 0.061 | 0.023 |

LTFU = lost at follow up
