# Supplementary material for: Putting in harm to cure: Drug related adverse events do not affect outcome of patients receiving treatment for multidrug-resistant Tuberculosis. Experience from a tertiary hospital in Italy
Source: PLoS One. 2019 Feb 28;14(2):e0212948. doi: 10.1371/journal.pone.0212948 (PMC6394924; doi:10.1371/journal.pone.0212948)
Supplement: S3 Table — (DOCX) [file pone.0212948.s003.docx]

**Supporting information tables**

**S3 Table. Correlation between outcome and AEs/SAEs**

| **Outcome** | **N.** | ***None AE*** | ***Total AE*** | ***SAE first 6 months*** | ***Total SAE (before and after six months)*** |
| --- | --- | --- | --- | --- | --- |
| **Cured** | 57 | 5 (8.8%) | 28 (49.1%) | 22 (38.6%) | 24 (42.1%) |
| **LTFU** | 15 | 3 (20%) | 8 (53.3%) | 4 (26.7%) | 4 (26.7%) |
| **Dead/failure** | 2 | 0 (0%) | 1 (50%) | 1 (50%) | 1 (50%) |
| p-value=0.832 | | | | | |

LTFU = lost at follow up
